# Preliminary Incidence and Trends of Infection with Pathogens Transmitted Commonly Through Food — Foodborne Diseases Active Surveillance Network, 10 U.S. Sites, 2006–2014

**Published:** 2015-05-15

**Authors:** Stacy M. Crim, Patricia M. Griffin, Robert Tauxe, Ellyn P. Marder, Debra Gilliss, Alicia B. Cronquist, Matthew Cartter, Melissa Tobin-D’Angelo, David Blythe, Kirk Smith, Sarah Lathrop, Shelley Zansky, Paul R. Cieslak, John Dunn, Kristin G. Holt, Beverly Wolpert, Olga L. Henao

**Affiliations:** 1 Division of Foodborne, Waterborne, and Environmental Diseases, National Center for Emerging and Zoonotic Infectious Diseases, CDC; 2 Atlanta Research and Education Foundation; 3 California Department of Public Health; 4 Colorado Department of Public Health and Environment; 5 Connecticut Department of Public Health; 6 Georgia Department of Public Health; 7 Maryland Department of Health and Mental Hygiene; 8 Minnesota Department of Health; 9 University of New Mexico; 10 New York State Department of Health; 11 Oregon Health Authority; 12 Tennessee Department of Health; 13 Food Safety and Inspection Service, US Department of Agriculture; 14 Center for Food Safety and Applied Nutrition, Food and Drug Administration

Foodborne illnesses represent a substantial, yet largely preventable, health burden in the United States. In 10 U.S. geographic areas, the Foodborne Diseases Active Surveillance Network* (FoodNet) monitors the incidence of laboratory-confirmed infections caused by nine pathogens transmitted commonly through food. This report summarizes preliminary 2014 data and describes changes in incidence compared with 2006–2008 and 2011–2013. In 2014, FoodNet reported 19,542 infections, 4,445 hospitalizations, and 71 deaths. The incidence of Shiga toxin–producing *Escherichia coli* (STEC) O157 and *Salmonella enterica* serotype Typhimurium infections declined in 2014 compared with 2006–2008, and the incidence of infection with *Campylobacter, Vibrio*, and *Salmonella* serotypes Infantis and Javiana was higher. Compared with 2011–2013, the incidence of STEC O157 and *Salmonella* Typhimurium infections was lower, and the incidence of STEC non-O157 and *Salmonella* serotype Infantis infections was higher in 2014. Despite ongoing food safety efforts, the incidence of many infections remains high, indicating that further prevention measures are needed to make food safer and achieve national health objectives.

FoodNet conducts active, population-based surveillance for laboratory-confirmed infections caused by *Campylobacter*, *Cryptosporidium*, *Cyclospora*, *Listeria*, *Salmonella*, Shiga toxin–producing *Escherichia coli* (STEC) O157 and non-O157, *Shigella*, *Vibrio*, and *Yersinia* in 10 geographic areas covering approximately 15% of the U.S. population (an estimated 48 million persons in 2013). FoodNet is a collaboration among CDC, 10 state health departments, the U.S. Department of Agriculture’s Food Safety and Inspection Service (USDA-FSIS), and the Food and Drug Administration (FDA). Hospitalizations occurring within 7 days of specimen collection are recorded, as is the patient’s vital status at hospital discharge or 7 days after specimen collection if the patient was not hospitalized. Hospitalizations and deaths that occur within 7 days of specimen collection are attributed to the infection. Surveillance for physician-diagnosed postdiarrheal hemolytic uremic syndrome, a complication of STEC infection characterized by renal failure, thrombocytopenia, and microangiopathic hemolytic anemia, is conducted through a network of nephrologists and infection preventionists and by hospital discharge data review. This report includes hemolytic uremic syndrome data for persons aged <18 years for 2013, the most recent year for which data are available.

Incidence was calculated by dividing the number of laboratory-confirmed infections in 2014 by U.S. Census estimates of the surveillance area population for 2013.† Incidence of culture-confirmed bacterial infections and laboratory-confirmed parasitic infections (e.g., identified by enzyme immunoassay) are reported. A negative binomial model with 95% confidence intervals (CIs) was used to estimate changes in incidence from 2006–2008 to 2014 and from 2011–2013 to 2014. Change in the combined overall incidence of infection with six key foodborne pathogens§ was estimated. For STEC non-O157, because of changing diagnostic practices and testing methods, only change in incidence since 2011–2013 was assessed; for *Cyclospora*, change was not assessed because data were sparse. For hemolytic uremic syndrome, 2013 incidence was compared with that in 2006–2008. The number of reports of positive culture-independent diagnostic tests without corresponding culture confirmation is reported for *Campylobacter*, *Salmonella*, *Shigella*, STEC, and *Vibrio*. Incidence calculations do not include culture-independent diagnostic test reports.

## Cases of Infection, Incidence, and Trends

In 2014, FoodNet identified 19,542 cases of infection, 4,445 hospitalizations, and 71 deaths (Table). The number and incidence per 100,000 population were as follows: *Salmonella* (7,452 [15.45]), *Campylobacter* (6,486 [13.45]), *Shigella* (2,801 [5.81]), *Cryptosporidium* (1,175 [2.44]), STEC non-O157 (690 [1.43]), STEC O157 (445 [0.92]), *Vibrio* (216 [0.45]), *Yersinia* (133 [0.28]), *Listeria* (118 [0.24]), and *Cyclospora* (26 [0.05]). The percentage of infections associated with outbreaks was as follows: STEC O157 (16%), *Listeria* (11%), STEC non-O157 (7%), *Shigella* (7%), *Salmonella* (6%), *Vibrio* (6%), *Cryptosporidium* (5%), *Yersinia* (0.8%), and *Campylobacter* (0.6%).

Among 6,565 (88%) serotyped *Salmonella* isolates in 2014, the number and incidence per 100,000 population of the top six serotypes were as follows: Enteritidis (1,401 [2.90]), Typhimurium (806 [1.67]), Newport (724 [1.50]), Javiana (639 [1.32]), I 4,[5],12:i:- (381 [0.79]), and Infantis (235 [0.49]). Among 208 (96%) speciated *Vibrio* isolates, 131 (63%) were *V. parahaemolyticus*, 27 (13%) were *V. alginolyticus*, and 19 (9%) were *V. vulnificus*. Among 546 (79%) serogrouped STEC non-O157 isolates, the top serogroups were O26 (31%), O103 (24%), and O111 (19%).

**FIGURE 1:**
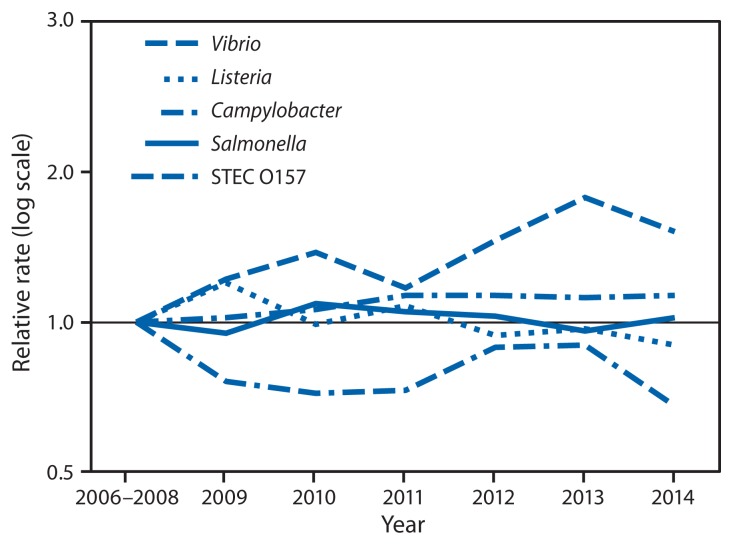
Relative rates of culture-confirmed infections with *Campylobacter*, STEC* O157, *Listeria*, *Salmonella*, and *Vibrio* compared with 2006–2008 rates, by year — Foodborne Diseases Active Surveillance Network, United States, 2006–2014^†^ * Shiga toxin–producing *Escherichia coli*. ^†^ The position of each line indicates the relative change in the incidence of that pathogen compared with 2006–2008. The actual incidences of these infections cannot be determined from this figure.

**FIGURE 2:**
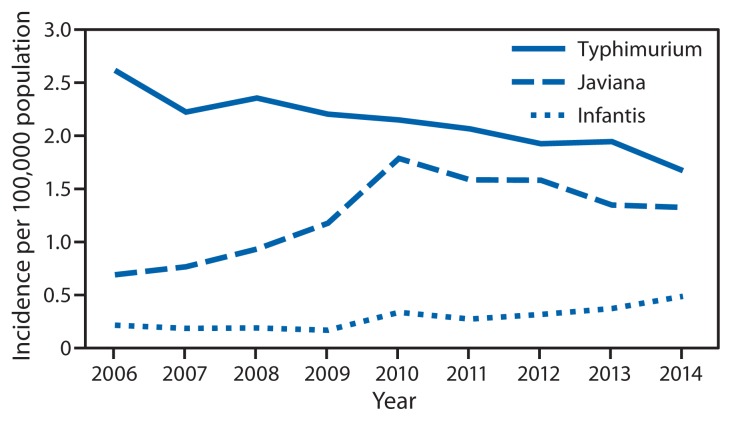
Incidence per 100,000 population of culture-confirmed infection with *Salmonella* serotypes Typhimurium, Javiana, and Infantis, by year — Foodborne Diseases Active Surveillance Network, United States, 2006–2014

Compared with 2006–2008, the 2014 incidence was significantly lower for STEC O157 (32% decrease; CI = 18%–43%) and *Yersinia* (22% decrease; CI = 1%–39%) infections, higher for *Vibrio* (52% increase; CI = 22%–89%) and *Campylobacter* (13% increase; CI = 5%–21%) infections, and not significantly changed for other pathogens (Figure 1). Among the six most commonly identified *Salmonella* serotypes, the incidence was significantly lower in 2014 for Typhimurium (27% decrease; CI = 18%–35%) compared with 2006–2008, but significantly higher for Infantis (162% increase; CI = 100%–244%) and Javiana (131% increase; CI = 83%–191%). Incidence for the three serotypes with significant changes in 2014 was calculated for the period 2006–2014 (Figure 2). Compared with 2011–2013, the 2014 incidence was significantly lower for STEC O157 and *Salmonella* serotype Typhimurium infections and higher for STEC non-O157 and *Salmonella* serotype Infantis infections. The overall incidence of infection with the six key foodborne pathogens was not significantly different from either of the comparison periods.

In 2013, a total of 87 cases of postdiarrheal hemolytic uremic syndrome were reported among children aged <18 years (0.79 cases per 100,000). Of these, 46 (53%) occurred in children aged <5 years (1.55 cases per 100,000). The incidence of hemolytic uremic syndrome was not significantly different than during 2006–2008 for either age group. No deaths were reported.

In addition to culture-confirmed infections (some with positive culture-independent diagnostic test results), there were 1,597 reports of positive culture-independent diagnostic tests that were not confirmed by culture, either because a culture did not yield the pathogen or because the specimen was not cultured. These reports were not included in the overall count of cases. Among 1,070 *Campylobacter* reports in this category, 553 (52%) had no culture, and 517 (48%) were culture-negative. Among 146 STEC reports, 62 (42%) had no culture, and 84 (58%) were culture-negative. The Shiga toxin–positive result was confirmed for 65 (48%) of 135 broths sent to a public health laboratory. The other reports of positive culture-independent diagnostic tests where culture was negative or not performed were of *Salmonella* (193), *Shigella* (186), and *Vibrio* (two).

### Discussion

In 2014, the incidence of laboratory-confirmed Shiga toxin–producing *E. coli* O157 and *Salmonella* serotype Typhimurium infections was significantly lower than during 2006–2008, whereas the incidence of *Campylobacter*, *Vibrio*, and *Salmonella* serotypes Javiana and Infantis infections was higher. Compared with 2011–2013, incidence of STEC non-O157 and *Salmonella* serotype Infantis infection was significantly higher.

The decrease in the incidence of STEC O157 infections could be attributable to several factors related to food safety efforts. Today, because isolates are routinely sent to public health departments for subtyping by PulseNet,¶ and epidemiologists rapidly investigate clusters of illnesses in which bacteria have similar DNA fingerprints, the sources of outbreaks are identified faster than in the past, which allows contaminated products to be removed from the marketplace before more persons become ill. The most common sources of STEC O157 infection are beef and leafy vegetables (1). After STEC O157 was declared an adulterant in ground beef in 1994, public health officials identified many STEC O157 outbreaks that resulted in ground beef recalls. Substantial changes in beef industry practices and government policy** led to a decrease in ground beef contamination (2). Contamination of ground beef with STEC O157 has decreased.†† Producers of leafy vegetables have also made improvements after a large outbreak in 2006 (3). It is also possible that a portion of the decrease is related to the increasing use of culture-independent diagnostic tests without confirmatory culture.

The increasing incidence of non-O157 STEC infections is attributable, in part, to an increase in the number of laboratories testing for Shiga toxin and, consequently, increased recognition of non-O157 STEC infections (4). Six serogroups (O26, O45, O103, O111, O121, and O145) are considered adulterants in non-intact beef products or the components of these products. In 2012, USDA-FSIS began testing for non-O157 STEC in domestic and imported beef manufacturing trimmings.§§

*Salmonella* serotypes are diverse in reservoirs and sources. The unchanged overall incidence of salmonellosis masks substantial changes in infection with individual serotypes. Typhimurium, the most common serotype reported to FoodNet until 2009, has contaminated a wide variety of food sources, including cattle and poultry. The incidence of Typhimurium infections nationwide has been declining since the mid-1980s, for reasons that are unclear (5). An analysis of outbreak data from 1998 to 2008 estimated that 34% of Typhimurium infections were related to consumption of poultry (1). Decreases in contamination of whole chickens with *Salmonella* serotype Typhimurium, as reported by USDA-FSIS¶¶ (Kristin Holt, USDA-FSIS; personal communication, 2013–2014 data, 2015), might have contributed to the decline. In July 2011, USDA-FSIS tightened the performance standards for *Salmonella* on poultry carcasses, and, in December 2013, released an action plan to decrease contamination in regulated products.*** Poultry vaccines against *Salmonella* have been used increasingly, first in egg-laying flocks, and to a lesser extent in broiler breeder flocks.††† *Salmonella* serotype Javiana infection is concentrated in southeastern states; the number of counties with annual infection rates above one case per 100,000 both inside and outside the southeast has increased markedly since the 1990s (5).

Additional regulations and ongoing industry efforts are likely to improve food safety. In January 2015, USDA-FSIS proposed new pathogen-reduction performance standards for *Salmonella* and *Campylobacter* in comminuted (reduced to minute particles) chicken and turkey products as well as raw chicken parts, such as chicken breasts, thighs, and wings.§§§ In 2015, FDA plans to publish regulations for safer produce, processed foods, and imported foods, as mandated by the Food Safety Modernization Act (6). Vaccination of breeder poultry flocks, in combination with biosecurity measures, has been shown to reduce contamination of poultry meat (7).

The findings in this report are subject to at least four limitations. First, increasing use of culture-independent tests by clinical laboratories might affect the number of culture-confirmed infections reported; culture-independent testing might increase (as observed for STEC non-O157 infections) or decrease (because fewer cases might be diagnosed through traditional methods) reported incidence (8). Second, health care–seeking behaviors and other characteristics of the population in the surveillance area might affect the generalizability of the findings. Third, the proportion of illnesses transmitted by nonfood routes differs by pathogen; data provided in this report are not limited to infections from food. Finally, changes in incidence between periods can reflect year-to-year variation during those periods rather than sustained trends, and the number of infections and patterns observed might change as final data become available.

Progress has been made in decreasing contamination of some foods and reducing illness caused by some pathogens. However, little or no recent reductions for most infections have occurred. For example, *Campylobacter* and *Vibrio* rates are still higher than during 2006–2008, a pattern also observed in 2013 (9). More information is needed to understand sources of infection and changes in incidence, and to help determine where to target prevention efforts.

What is already known on this topic?

The incidence of many foodborne infections, including *Salmonella*, has been largely unchanged for many years. *Salmonella* infection is a complicated problem because infection can be acquired by many types of foods and from nonfood sources, so many different control methods are needed.

What is added by this report?

The 2014 data show progress in reducing infections from *Escherichia coli* O157 and *Salmonella* serotype Typhimurium. However, the incidence of infection with *Salmonella* serotypes Infantis and Javiana has increased.

What are the implications for public health practice?

Infections caused by *E. coli* serogroup O157 declined after targeted interventions to reduce contamination of ground beef were implemented. Similarly, to reduce the incidence of *Salmonella* infection, serotype-specific approaches are required. Public health, regulatory agencies, industry, and consumers can all play a role.

## Figures and Tables

**TABLE:** Number of cases of culture-confirmed bacterial and laboratory-confirmed parasitic infection, hospitalizations, and deaths, by pathogen — Foodborne Diseases Active Surveillance Network, United States, 2014*

Pathogen	Cases			Hospitalizations		Deaths	
		
	No.	Incidence†	Objective§	No.	(%)	No.	(%)
**Bacteria**							
*Campylobacter*	6,486	13.45	8.5	1,080	(17)	11	(0.2)
*Listeria*	118	0.24	0.2	108	(92)	18	(15.3)
*Salmonella*	7,452	15.45	11.4	2,141	(29)	30	(0.4)
*Shigella*	2,801	5.81	N/A¶	569	(20)	2	(0.1)
STEC O157	445	0.92	0.6	154	(35)	3	(0.7)
STEC non-O157	690	1.43	N/A	104	(15)	0	(0.0)
*Vibrio*	216	0.45	0.2	40	(19)	2	(0.9)
*Yersinia*	133	0.28	0.3	30	(23)	1	(0.8)
**Parasites**							
*Cryptosporidium*	1,175	2.44	N/A	217	(18)	4	(0.3)
*Cyclospora*	26	0.05	N/A	2	(8)	0	(0.0)
**Total**	**19,542**			**4,445**		**71**	

**Abbreviations:** N/A = not available; STEC = Shiga toxin–producing *Escherichia coli.*

* Data for 2014 are preliminary.

† Per 100,000 population.

§ *Healthy People 2020* objective targets for incidence of *Campylobacter*, *Listeria*, *Salmonella*, STEC O157, *Vibrio*, and *Yersinia* infections per 100,000 population.

¶ No national health objective exists for these pathogens.

## Footnotes

* Additional information available at http://www.cdc.gov/foodnet.

† Final incidence rates will be reported when population estimates for 2014 are available.

§ The overall incidence of infection combines data for *Campylobacter*, *Listeria*, *Salmonella*, STEC O157, *Vibrio*, and *Yersinia*, six key bacterial pathogens for which >50% of illnesses are estimated to be transmitted by food.

¶ PulseNet is the national molecular subtyping network for foodborne bacterial pathogens.

** The Federal Register notice is available at http://www.fsis.usda.gov/wps/wcm/connect/ed6d4959-a499-4e6e-af86-ae8a419ba156/00-022N.htm?MOD=AJPERES.

†† Data on ground beef contamination by year are available at http://www.fsis.usda.gov/wps/portal/fsis/topics/data-collection-and-reports/microbiology/ec/positive-results-current-cy/positive-results-current-cy.

§§ More information is available at http://www.fsis.usda.gov/wps/portal/frameredirect?url=http://www.fsis.usda.gov/OPPDE/rdad/FRPubs/2010-0023FRN.htm.

¶¶ FSIS serotype report through 2012 available at http://www.fsis.usda.gov/wps/wcm/connect/180fc804-0311-4b4d-ae42-d735e8232e1c/Salmonella-Serotype-Annual-2012.pdf?MOD=AJPERES.

*** More information is available at http://www.fsis.usda.gov/wps/wcm/connect/aae911af-f918-4fe1-bc42-7b957b2e942a/SAP-120413.pdf?MOD=AJPERES.

††† More information is available at https://www.avma.org/News/JAVMANews/Pages/101215x.aspx.

§§§ The Federal Register notice is available at http://www.fsis.usda.gov/wps/wcm/connect/b711839a-c0b9-420f-9d74-8568310a1352/2014-0023.htm?MOD=AJPERES.
